# Supplementary material for: Netcombin: An algorithm for constructing optimal phylogenetic network from rooted triplets
Source: PLoS One. 2020 Sep 18;15(9):e0227842. doi: 10.1371/journal.pone.0227842 (PMC7500971; doi:10.1371/journal.pone.0227842)
Supplement: S4 File — (ZIP) [file pone.0227842.s004.zip › SequenceData-Triplet/sequenceData-10-20-40-Triplet2/data-(sample 10- length 500- num seq 40- m rate 10^-6)/10 seq.pdf]

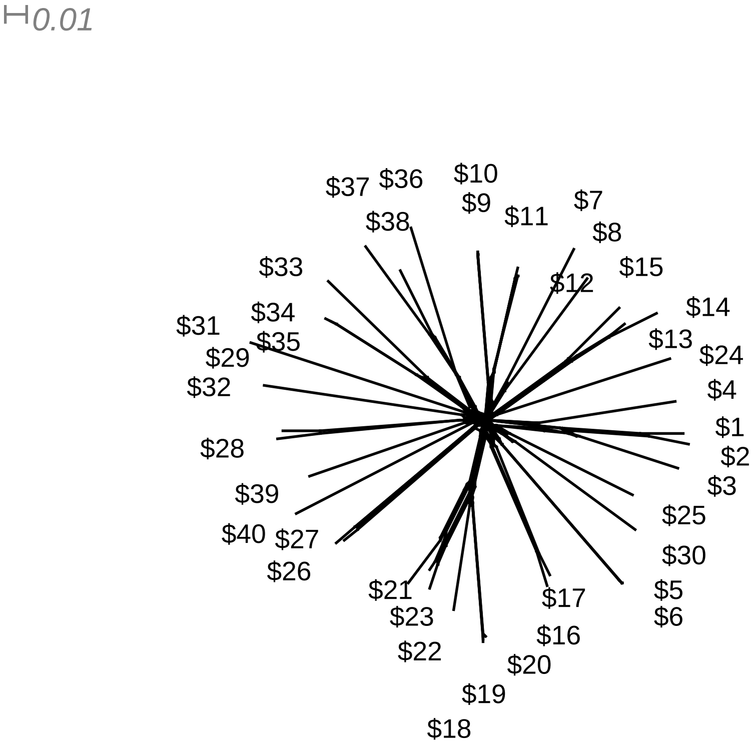

0.01
$37
$36
$10
$9
$11
$7
$8
$38
$33
$12
$15
$14
$34
$31
$35
$13
$29
$24
$32
$4
$1
$28
$2
$3
$39
$25
$40
$27
$30
$26
$5
$21
$17
$6
$23
$16
$22
$20
$19
$18
